# Identification of potential diagnostic biomarkers of atherosclerosis based on bioinformatics strategy

**DOI:** 10.1186/s12920-023-01531-w

**Published:** 2023-05-12

**Authors:** Zhipeng Zheng, Dong Yuan, Cheng Shen, Zhiyuan Zhang, Jun Ye, Li Zhu

**Affiliations:** 1 Dalian Medical University, Dalian, 116000 China; 2 Nanjing University of Chinese Medicine, Nanjing, 210023 China; 3 The Affiliated Taizhou People’s Hospital of Nanjing Medical University, Taizhou, 225300 China

**Keywords:** Atherosclerosis, Gene expression profiling, Robust rank aggregation, Hub genes, Bioinformatics

## Abstract

**Background:**

Atherosclerosis is the main pathological change in atherosclerotic cardiovascular disease, and its underlying mechanisms are not well understood. The aim of this study was to explore the hub genes involved in atherosclerosis and their potential mechanisms through bioinformatics analysis.

**Methods:**

Three microarray datasets from Gene Expression Omnibus (GEO) identified robust differentially expressed genes (DEGs) by robust rank aggregation (RRA). We performed connectivity map (CMap) analysis and functional enrichment analysis on robust DEGs and constructed a protein‒protein interaction (PPI) network using the STRING database to identify the hub gene using 12 algorithms of cytoHubba in Cytoscape. Receiver operating characteristic (ROC) analysis was used to assess the diagnostic potency of the hub genes.The CIBERSORT algorithm was used to perform immunocyte infiltration analysis and explore the association between the identified biomarkers and infiltrating immunocytes using Spearman’s rank correlation analysis in R software. Finally, we evaluated the expression of the hub gene in foam cells.

**Results:**

A total of 155 robust DEGs were screened by RRA and were revealed to be mainly associated with cytokines and chemokines by functional enrichment analysis. CD52 and IL1RN were identified as hub genes and were validated in the GSE40231 dataset. Immunocyte infiltration analysis showed that CD52 was positively correlated with gamma delta T cells, M1 macrophages and CD4 memory resting T cells, while IL1RN was positively correlated with monocytes and activated mast cells. RT-qPCR results indicate that CD52 and IL1RN were highly expressed in foam cells, in agreement with bioinformatics analysis.

**Conclusions:**

​This study has established that CD52 and IL1RN may play a key role in the occurrence and development of atherosclerosis, which opens new lines of thought for further research on the pathogenesis of atherosclerosis.

**Supplementary Information:**

The online version contains supplementary material available at 10.1186/s12920-023-01531-w.

## Introduction

Atherosclerotic cardiovascular disease (ASCVD) remains the leading cause of death worldwide and is a major component of the world disease burden. Atherosclerosis is the main pathological change in ASCVD, and often causes ischaemic heart disease, ischaemic stroke, peripheral artery disease and other major diseases [1–4]. Despite considerable efforts to elucidate the cellular and molecular mechanisms underlying atherosclerosis, its complex mechanisms are still not fully understood [5, 6]. Therefore, an in-depth exploration of the pathological mechanisms of atherosclerosis is of great research and clinical importance for the prevention and treatment of ASCVD.

In recent years, bioinformatics analysis has become an important method in modern medical science research. GEO public database has been widely used to explore candidate genes and new mechanisms of different diseases [7–9]. Therefore, the exploration of transcriptome data through bioinformatics analysis can tap into some potential mechanisms of atheroma to provide new diagnostic biomarkers and contribute to future clinical research.

**Fig. 1:**
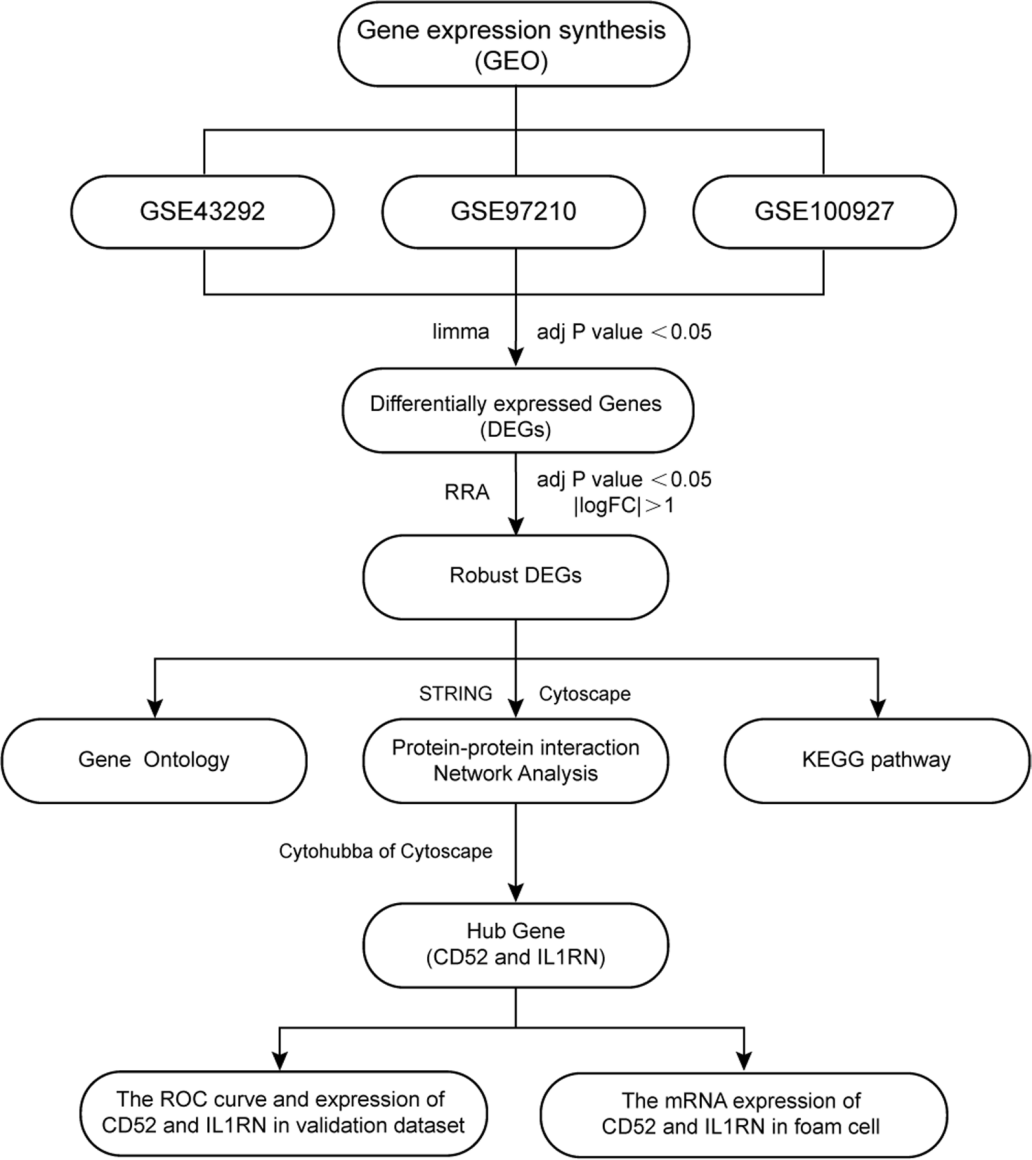
Flowchart of the research

In this study, we aimed to explore the underlying mechanisms of atherosclerosis and identify clinically valuable diagnostic biomarkers through comprehensive bioinformatics analysis. To minimize the impact of different laboratories, technical platforms, and different data processing methods, we downloaded three microarray datasets of atherosclerosis from GEO and obtained integrated differentially expressed genes (DEGs) using the robust rank aggregation (RRA) method. Subsequently, we performed connectivity map (CMap) analysis, gene ontology (GO) and Kyoto Encyclopedia of Genes and Genomes (KEGG) enrichment analysis of the DEGs and constructed a PPI network using the STRING database to identify the hub gene using the 12 algorithms of cytoHubba in Cytoscape. We assessed the diagnostic power of the hub genes by Receiver operating characteristic (ROC) analysis, performed immunocyte infiltration analysis with the use of the CIBERSORT algorithm, and explored the association between the identified biomarkers and infiltrating immunocytes using Spearman’s rank correlation analysis in R software. Finally, we evaluated the expression levels of the hub genes in foam cells. A flowchart summarizing this study is shown in Fig. 1.

## Materials and methods

### Collection and pretreatment of microarray data

Microarray gene expression datasets were retrieved from the GEO database (https://www.ncbi.nlm.nih.gov/gds) for atherosclerosis and matched controls. We used “atherosclerosis” as the key word for retrieval in the GEO database, and selected the atherosclerotic microarray dataset according to the following criteria: (1) samples of human atherosclerotic vascular tissue and normal vascular tissue, (2) gene expression profile of mRNA, (3) at least three samples per group, (4) expression profiling by array as the study type. Based on the above screening criteria, four datasets were eventually included in the study: GSE43292, GSE97210, GSE100927, and GSE40231. Of these, GSE43292, GSE97210, GSE100927 were used for joint analysis and identification of hub genes, and GSE40231 was used as a validation set to assess the diagnostic efficacy of hub genes. The fundamentals of these microarray datasets were listed in Additional file 1: Table S1. In the R (https://www.r-project.org/versionr-4.1.1), the “arrayQualityMetrics” package was used for quality control of samples in the dataset, and the “affy” or “the linear models for microarray data (limma)” packages were used to standardize presentation data. The values of DEGs, adjusted *p* value < 0.05 and | log2 Fold change (logFC) | >1 between atherosclerosis and control samples in each dataset were identified as statistically significant by the “limma” packet in R. Volcanic and thermal maps were plotted using the “ggplot2”package and the “pheatmap” package in R, respectively.

### Robust rank aggregation analysis

To integrate the DEGs of the three microarray data sets, we ranked the up-regulation and down-regulation genes for |logFC| >1.0 in each dataset by their logFC and then determined the robust DEGs using the “RobustRankAggreg” packet in R. RRA is a standard method of minimizing deviations and errors between multiple datasets [10]. Genes with |logFC| >1.0 and *p* < 0.05 were considered significant robust DEGs. The heatmap is drawn using the “pheatmap” package in R.

The “clusterProfiler” package in R was used to perform the GO and KEGG pathway enrichment analyses for robust DEGs [11–13]. The GO term and the KEGG pathway that met both p-value < 0.05 and q-value < 0.05 were considered to be statistically significant enrichment. The package ‘ggplot2’ in R was used to visualize the results of these enrichment analyses.

### Connectivity map (CMap) analysis

Robust DEGs were analyzed using the CMap database (https://clue.io/) to explore potential anti-atherosclerosis agents [14]. CMap is an online database that can analyze the characteristics of gene expression profiles of diseases and find small molecule compounds with similar or opposite effects, which is often used to predict potential therapeutic drugs for diseases.

### Construction of PPI networks and identification of hub genes

The PPI network of robust DEGs was constructed in the STRING online database (https://string-db.org/, version 11.5) using default parameters, and the results were imported into the Cytoscape software (http://cytoscape.org/; version 3.7.2) for visualization [15]. The hub genes were then identified using the 12 algorithms of cytoHubba in Cytoscape software, and visualization was performed using the “UpSetR” package in R.

### Diagnostic value of hub genes in atherosclerosis

The ROC curve was generated by the “pROC” packet in R to evaluate the diagnostic efficacy of the identified hub genes in the GSE43292, GSE97210, and GSE100927 datasets (27). The area under the ROC curve (AUC) value was used to determine the diagnostic effectiveness of distinguishing between atherosclerosis and the control sample. These hub genes were further validated in the GSE40231 dataset. The “ggpubr” in R was used to visualize differences in the expression of hub genes in the GSE40231 dataset.

### Immune Infiltration by CIBERSORT Analysis

The CIBERSORT algorithm performed immunocyte infiltration analysis on the GSE40231 dataset [16]. The levels of 22 immune cells between the two groups were compared using the “vioplot” package, and the “corrplot” package was used to make the correlation heatmap of 22 immune cells. The Spearman rank correlation analysis in R software explored the association between characteristic genes and infiltrating immune cells, and visualized it using the “ggplot2” package. *P* < 0.05 were considered statistically significant.

### Cell culture and treatment

Human THP-1 cells (CL-0233) were kindly provided by Procell Life Science&Technology (Wuhan, China) and subjected to STR authentication in Procell Life Science&Technology. THP-1 cells were cultured with RPMI-1640 (G4530-500ML, Servicebio, China) supplemented with 10% Fetal Bovine Serum (FBS) (FSP500, ExCell Bio, China) and 1% Penicillin-Streptomycin (P/S) (15,140,122, GIBCO, USA). The cells were inoculated in 6-well plates at a density of 5 * 10^5/ml, and the cells were inoculated with phorbol 12-myristate 13-acetate (PMA) (100 ng/ml; Hy-18,739, MedChemExpress, USA) was cultured in the culture medium for 48 h to differentiate into macrophages. THP-1 macrophages differentiated from PMA were treated with 80 µg/ml ox-LDL (Yiyuan Biotechnology, China) for 24 h. Foam cell development was determined using Oil Red O staining. An incubator with 5% CO_2_ and 37 °C was used to culture cells.

### Oil red O staining

Each well of the medium was sucked out, and the cells were washed gently with PBS for 3 to 5 times, fixed with 4% paraformaldehyde at 37℃ for 30 min, rinsed with PBS twice, and stained with oil red O (00625, Sigma-Aldrich, USA) at 37℃ for 30 min. Rinse with 60% 2-propanol for 5s, and then 2–3 times with PBS. The lignin was counterstained for 20-60s and immediately rinsed 2–3 times with PBS. Oil red O staining was observed under a microscope. Measuring oil red positive area with image J software.

### Real-time quantitative PCR (RT-qPCR)

Total RNA was extracted with E.Z.N.A Total RNA Kit I (R6834, OMEGA, USA) and cDNA synthesis was performed by the All-In-One 5X RT MasterMix (G592, abm, Canada) according to the manufacturer’s instructions. LightCycler 480 II System (Roche, Switzerland) and ChamQ Universal SYBR qPCR Master Mix (Q711, Vazyme, China) were used for quantitative real-time PCR analysis. Data were normalized to β-ACTIN expression in each sample. The specific primers used for RT-qPCR were shown in Table 1.

**Table 1:** Primers used for RT-qPCR

Gene	Forward (5′–3′)	Reverse (5′–3′)
β-Actin	CATGTACGTTGCTATCCAGGC	CTCCTTAATGTCACGCACGAT
CD52	TCTTCCTCCTACTCACCATCAG	CCTCCGCTTATGTTGCTGGA
IL1RN	CATTGAGCCTCATGCTCTGTT	CGCTGTCTGAGCGGATGAA

### Statistical analysis

In vitro validation data were analyzed using Prism 9.0 (GraphPad, La Jolla, CA, USA). Data were expressed as the mean standard deviation (SD) of not less than three independent experiments, and differences were analyzed using t-test (two groups). The dataset was analyzed by R software (version 4.1.1), and the difference was analyzed by Wilcoxon test (two groups) and ANOVA (more than two groups). Significant differences were considered to be *p* < 0.05 unless otherwise noted.

## Results

### Identification of DEGs in each dataset

**Fig. 2:**
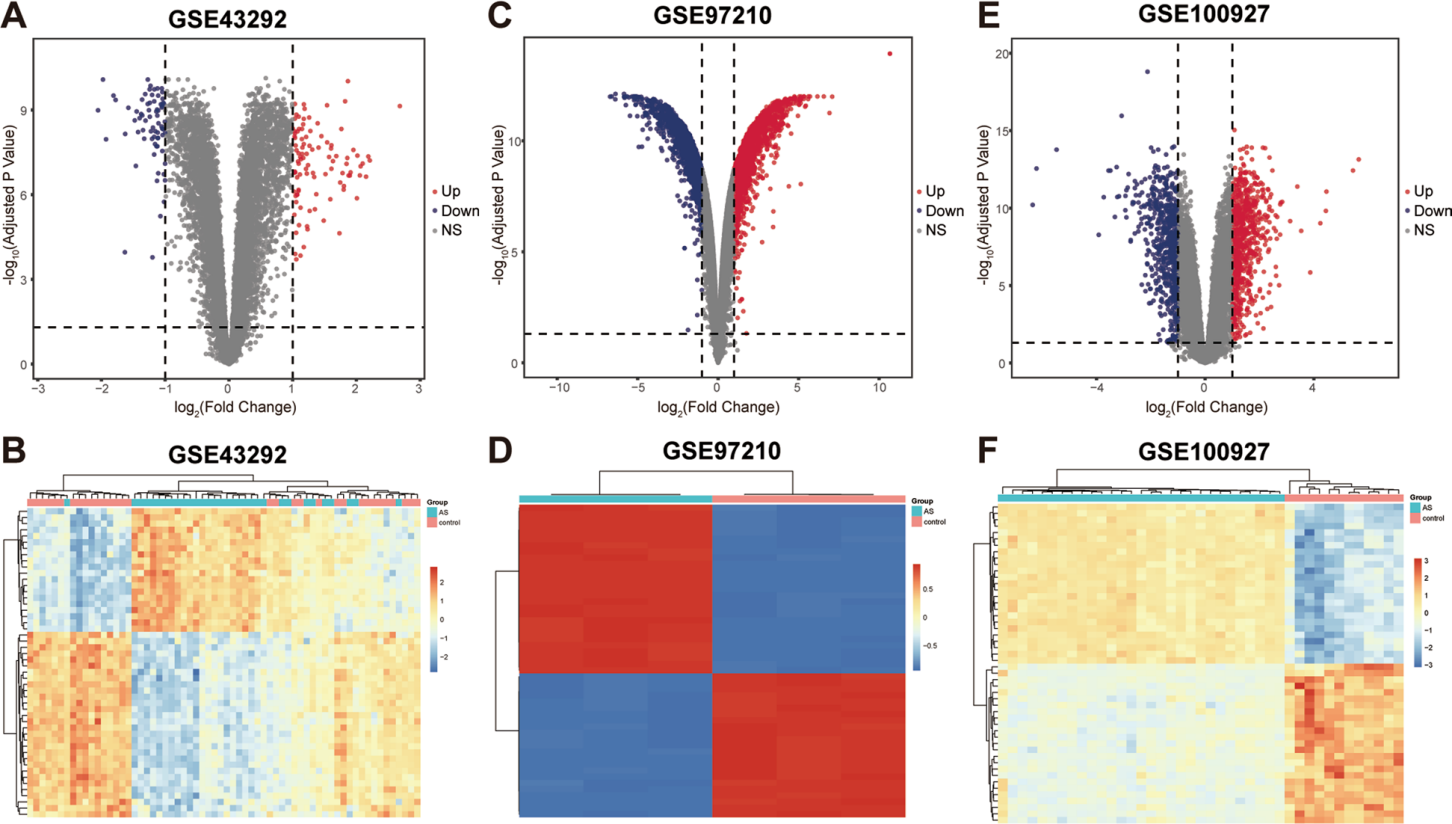
Volcano plot and heatmap of differential expression of GSE43292 (**A, B**), GSE97210 (**C, D**), and GSE100927 (**E, F**). In the volcano plot, the blue plots represent downregulated DEGs; the gray plots represent non-significant genes, and the red plots represent upregulated DEGs. The heatmap shows the two-way hierarchical clustering results of the top 50 ranked differential expression profiles and samples with adjusted *P* values. Each row in the heatmap represents a gene, and each column represents a sample. The color scale on the right side of the heatmap represents the homogenous expression value, ranging from blue (low expression) to red (high expression)

After normalization and analysis using the “limma” package in R according to predetermined thresholds, we screened out 174 DEGs (101 upregulated and 73 downregulated) in GSE43292, 2370 upregulated DEGs and 2344 downregulated DEGs from GSE97210. Meanwhile, we also identified 761 up-regulated DEGs and 645 down regulated DEGs from GSE100927 (Additional file 2: Table S2). Volcano plots present the differential expression results for the microarray dataset (Fig. 2A C, E), and the top 50 ranked differential expression data with adjusted *P* value were visualized in heatmap (Fig. 2B, D and F).

### Identification of robust DEGs by RRA method

**Fig. 3:**
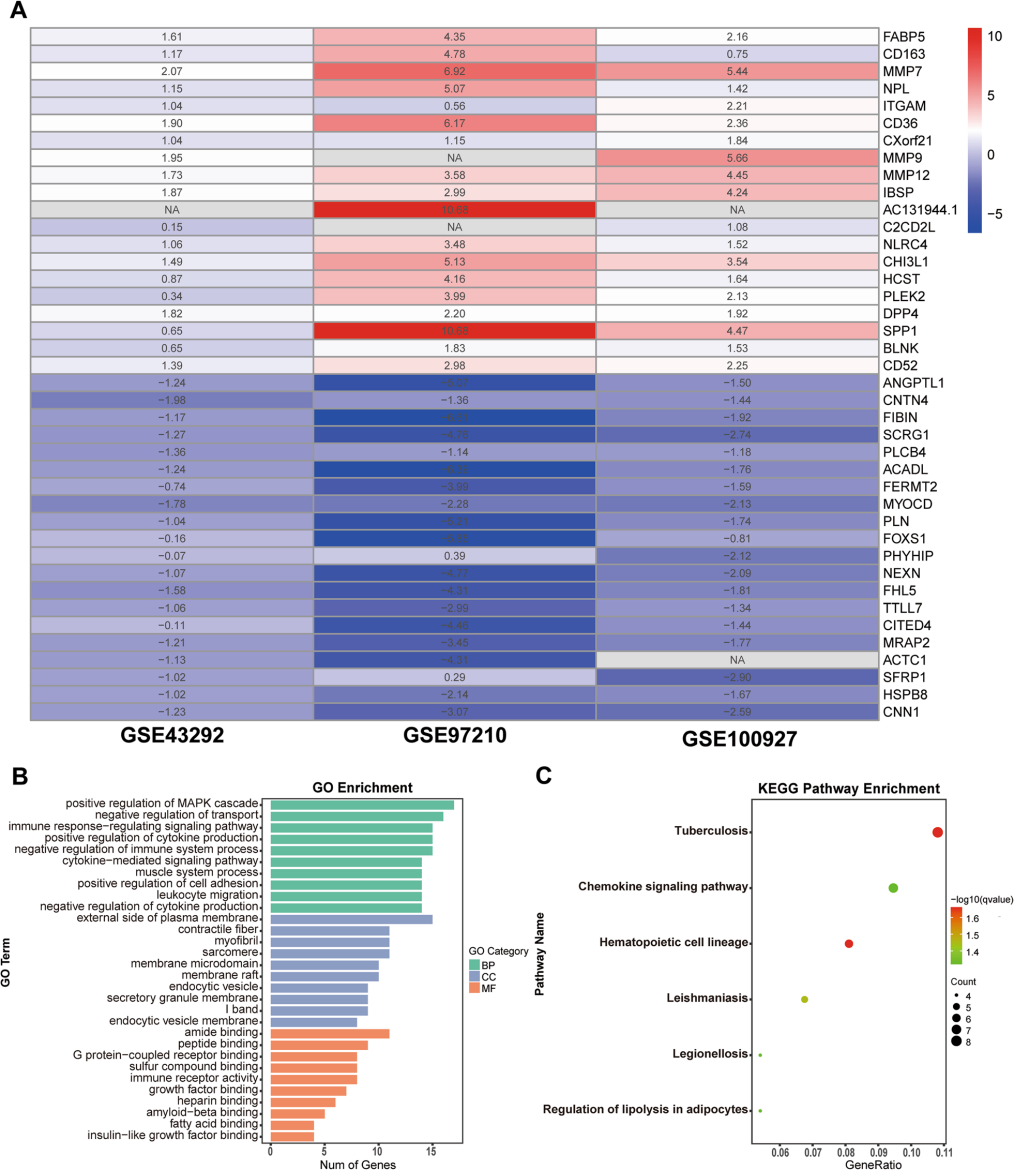
Identification and functional enrichment analysis of robust DEGs. **A** Robust DEGs for 3 microarray datasets were identified and integrated using the RRA. The rows represent the genes and the columns represent the GEO dataset. The color of the heatmap represents the homogenous expression value, ranging from blue (low expression) to red (high expression), and the value in the box represents the logFC value. NA means that the gene was not statistically significant in this dataset. **B **GO functional enrichment of robust DEGs, BP (biological processes), CC (cellular components), MF (molecular functions). **C** KEGG pathway enrichment of robust DEGs

We integrated the three data sets using the RRA method and determined a total of 155 robust DEGs, including 76 up-regulated and 79 down-regulated genes (Additional file 3: Table S3). Based on the p-value of the robust DEGs, we visualized the expression of the top 40 robust DEGs (top 20 up-regulated and top 20 down-regulated genes) from the RRA analysis using the “pheatmap” package in R (Fig. 3A).

### Functional Enrichment analysis of robust DEGs

The GO functional enrichment analysis results of 155 robust DEGs were 555, including 524 biological processes (BP), 18 cellular components (CC), and 13 molecular functions (MF) (Additional file 4: Table 4). The GO enrichment analysis (Fig. 3B) revealed significantly enriched BP in cytokine regulation, immune and inflammation regulation, and MAPK cascade regulation. For CC terminology, robust DEGs were mainly enriched in contractile fibers, myofibrils, sarcomere, endocytic vesicle, endocytic vesicle membrane, secretory granule membrane, etc. In addition, growth factor binding, immune receptor activity, amide binding, peptide binding, G protein-coupled receptor binding, fatty acid binding, and others were significantly enriched in MF. Meanwhile, KEGG pathway analysis showed (Fig. 3C) that the 155 robust DEGs were significantly enriched in six pathways, such as chemokine signaling pathway, hematopoietic cell lineage, and regulation of lipolysis in adipocytes (Additional file 5: Table S5).

### CMap analysis

To search for potential small molecule compounds to reverse robust DEGs expression profiles, CMap analysis was performed. We showed the most significant top 10 negatively correlated drugs/molecules, including KU-C103428N, desoxypeganine, MBCQ, phylloquinone, gossypol, kavain, flubendazole, VX-702, palonosetron, linsitinib (Table 2).

**Table 2:** List of the 10 most important small molecule compounds provided by CMap analysis based on robust DEGs

CMap name	Description	Target	Score
KU-C103428N	CDC inhibitor	NFE2	− 95.01
Desoxypeganine	Acetylcholinesterase inhibitor	ACHE, MAOA	− 91.59
MBCQ	Phosphodiesterase inhibitor	PDE5A	− 90.16
Phylloquinone	Vitamin K	BGLAP, GGCX	− 90.11
Gossypol	BCL inhibitor	BCL2, BCL2L1, MCL1, BCL2L2, CTGF, EGF	− 89.72
Kavain	Calcium channel modulator	MTOR	− 89.71
Flubendazole	Tubulin inhibitor	TUBB	− 86.28
VX-702	p38 MAPK inhibitor	MAPK14, MAPK11, MAPK12	− 85.43
Palonosetron	Serotonin receptor antagonist	HTR3A	− 83.07
Linsitinib	IGF-1 inhibitor	IGF1R, INSR, INSRR	− 81.75

### PPI Network Construction and hub genes identification

**Fig. 4:**
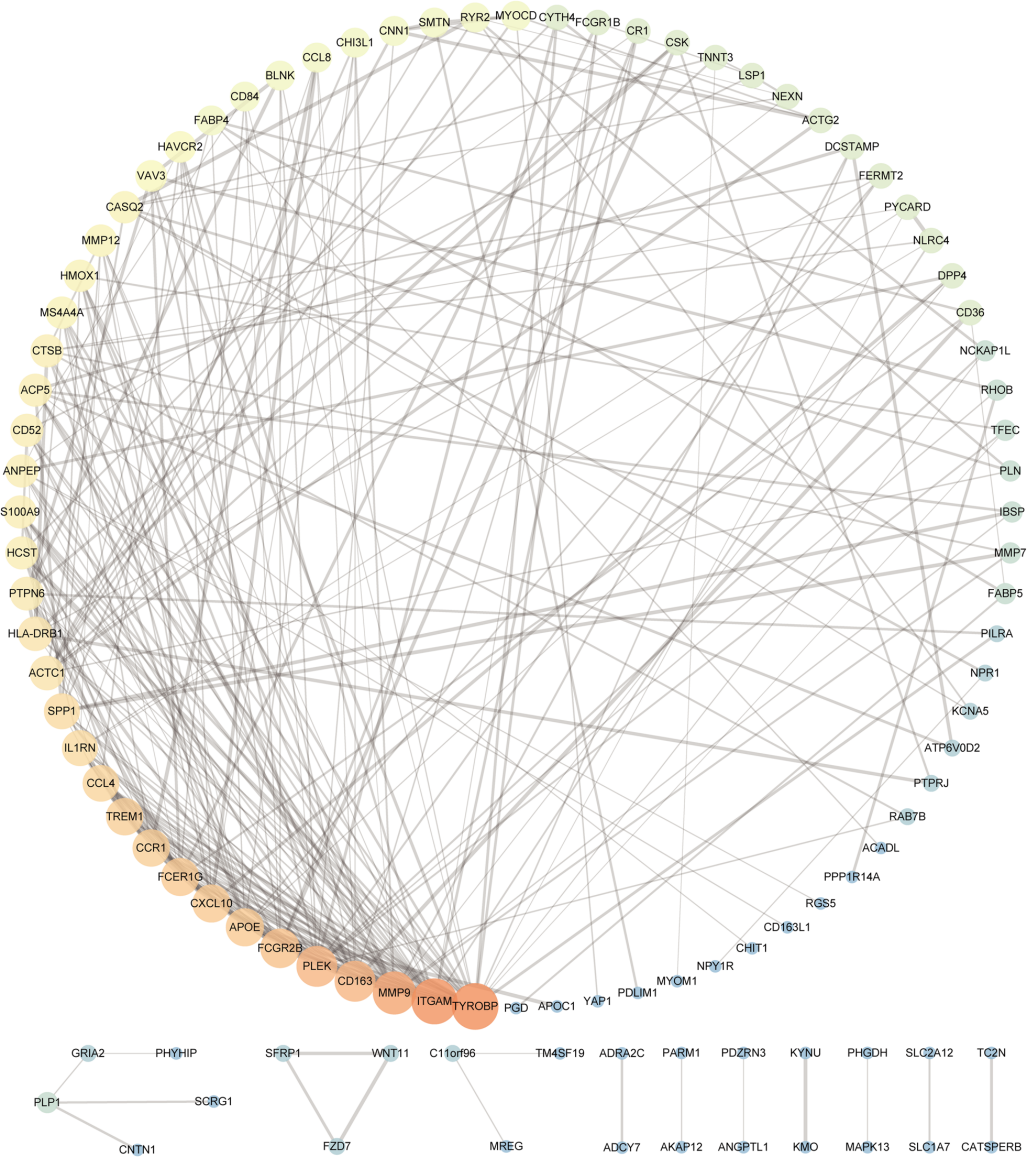
PPI network for robust DEGs. A larger shape and red color of the node indicated that the “Degree” weight of the gene was larger. Conversely, a smaller shape and blue color indicated that the node had a larger degree weight. The thickness of the line between nodes represents the value of “Conbined_Score” between genes, and a thicker line represents a larger value

**Fig. 5:**
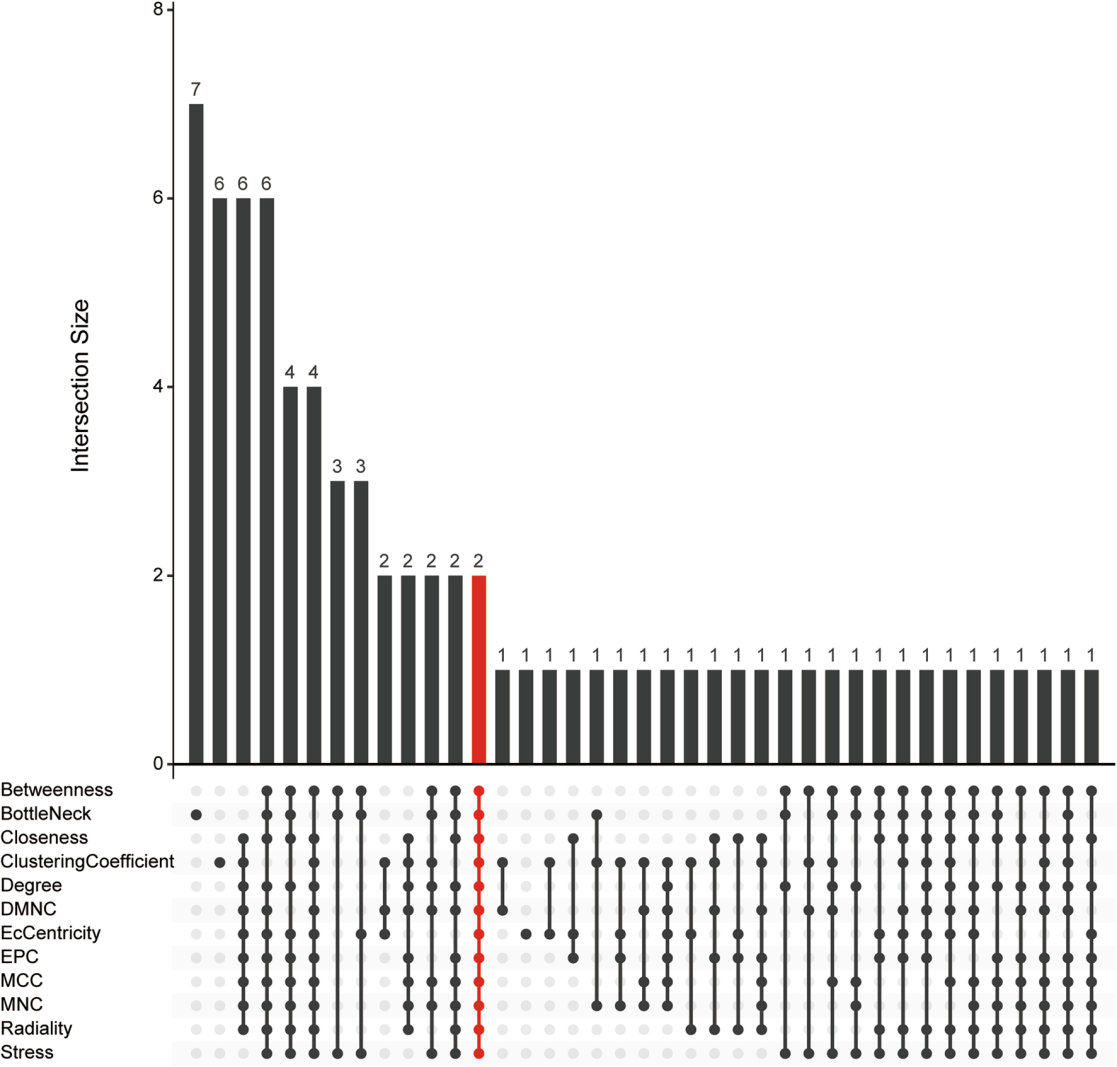
The 12 algorithms of cytoHubbain Cytoscape software identify the hub gene in the PPI network and are visualized by the “UpSet” package in R. The abscissa represents the overlapping mode of different algorithms, and the ordinate represents the number of genes overlapped by different algorithms. The section marked red shows the overlapping results of the 12 algorithms of cytoHubba

To further investigate the interaction of robust DEGs, we constructed a PPI network using a STRING database with the confidence > 0.9 and hidden disconnected nodes, and visualized it on Cytoscape software (Fig. 4). In the final network, there were 101 nodes and 267 edges. Then, we got top 40 robust DEGs from protein-protein network ranked by 12 different algorithms of cytoHubbaincluding Degree, Density of Maximum Neighborhood Component (DMNC), Edge Percolated Component (EPC), Maximal Clique Centrality (MCC), Maximum Neighborhood Component (MNC), and centralities based on shortest paths, such as Bottleneck (BN), Closeness, EcCentricity (EC), Radiality, Betweenness, Stress, and Clustering Coefficient (CC) were intersected. Finally, CD52 and IL1RN were identified as hub genes (Fig. 5).

## Clinical diagnostic significance of potential biomarkers for atherosclerosis

**Fig. 6:**
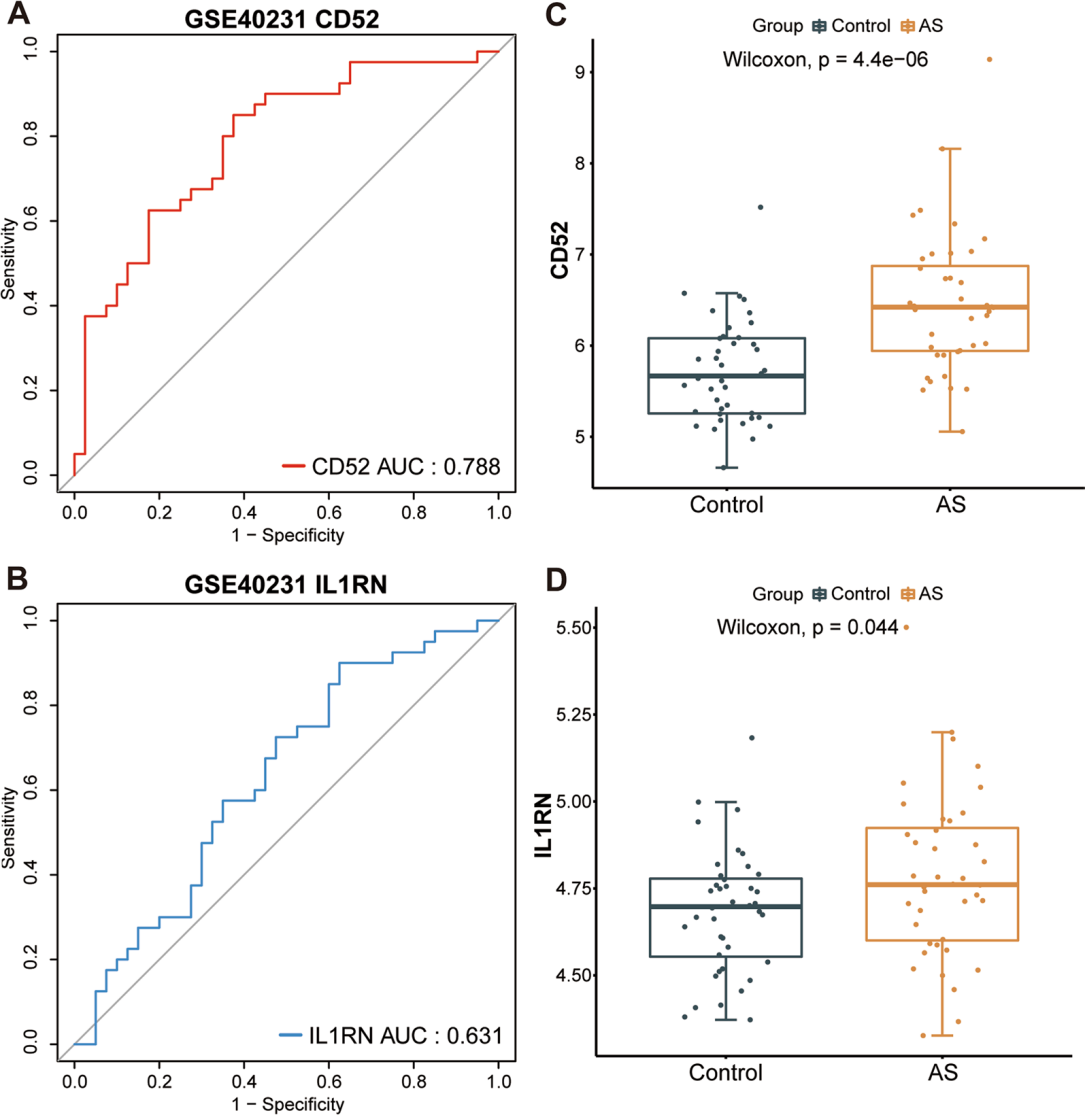
Validation of biomarkers of atherosclerosis in the GSE40231 dataset. **A**, **B** ROC curve evaluation of the diagnostic effectiveness of candidate biomarkers using the GSE40231 dataset. **C**, **D** Expression of candidate diagnostic biomarkers in the GSE40231 dataset

The ROC curve suggests that CD52 and IL1RN showed favorable diagnostic value in distinguishing between atherosclerosis and control data in the GSE43292, GSE97210, and GSE100927 datasets (Additional file 9: Figure S1 A-F). To further validate the diagnostic value of the above genes, we demonstrated their satisfactory discrimination in the GSE40231 validation dataset, with an AUC of 0.788 in CD52 and 0.631 in IL1RN (Fig. 6A and B). In addition, expression levels of CD52 and IL1RN in the atherosclerosis samples were also significantly higher than in the normal samples (Fig. 6C and D).

### Immune infiltration analyses

**Fig. 7:**
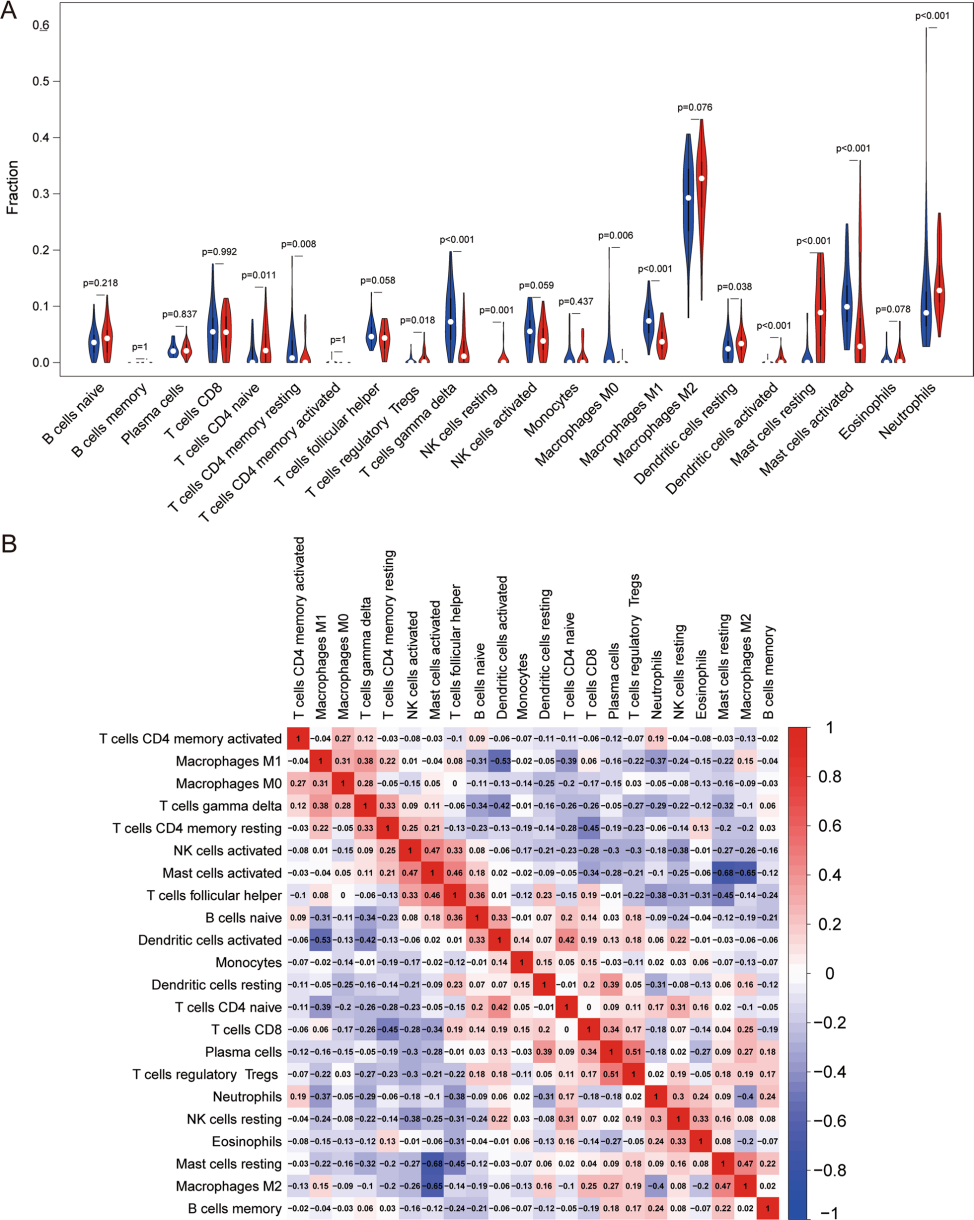
Distribution and visualization of immune cell infiltration. **A** Comparison of 22 immune cell subtypes between atherosclerosis tissues and normal tissues. Blue and red colors indicate normal and atherosclerotic samples, respectively. **B** An associated matrix of all 22 immune cell subtypes. Both the horizontal and vertical axes show immunocyte subtypes. Immunocyte subtype compositions (higher, lower, and same correlation levels are displayed in red, blue, and white, respectively; the values in the boxes represent the correlation coefficient)

Immunocyte infiltration analysis on the GSE40231 dataset showed that the proportions of CD4 memory resting T cells, gamma delta T cells, M0 macrophages, M1 macrophages and activated mast cells in atherosclerosis tissue were significantly lower than those in normal tissue. However, the proportion of CD4 naive T cells, regulatory Tregs T cells, activated dendritic cells, resting mast cells and neutrophils in atherosclerosis tissue was significantly higher than that in normal tissue (Fig. 7A). The correlation heatmap (Fig. 7B) related to cell-type abundances showed a significant negative correlation between CD4 memory resting T cells and CD8 T cells, naive B cells, and CD4 naive T cells. Regulatory Tregs T cells showed a significant positive correlation with plasma cells and a negative correlation with CD4 memory resting T cells and follicular helper T cells. Gamma delta T cells were positively correlated with CD4 memory resting T cells, M0 macrophages, and M1 macrophages, and negatively correlated with naive B cells. M0 macrophages were positively correlated with CD4 memory activated T cells and M1 macrophages. M1 macrophages were negatively correlated with CD4 naive T cells and naive B cells. Activated dendritic cells were positively correlated with CD4 naive T cells and naive B cells, and negatively correlated with M1 macrophages and gamma delta T cells. Resting mast cells were positively correlated with M2 macrophages, and negatively correlated with follicular helper T cells and gamma delta T cells. Activated mast cells were positively correlated with activated NK cells, follicular helper T cells, and negatively correlated with M2 macrophages and CD8 T cells. neutrophils and resting NK cells were positively correlated, and M2 macrophages, follicular helper T cells, and M1 macrophages were negatively correlated (Additional file 6, 7: Tables S6 and S7).

### The correlation between hub genes and Immune cells

**Fig. 8:**
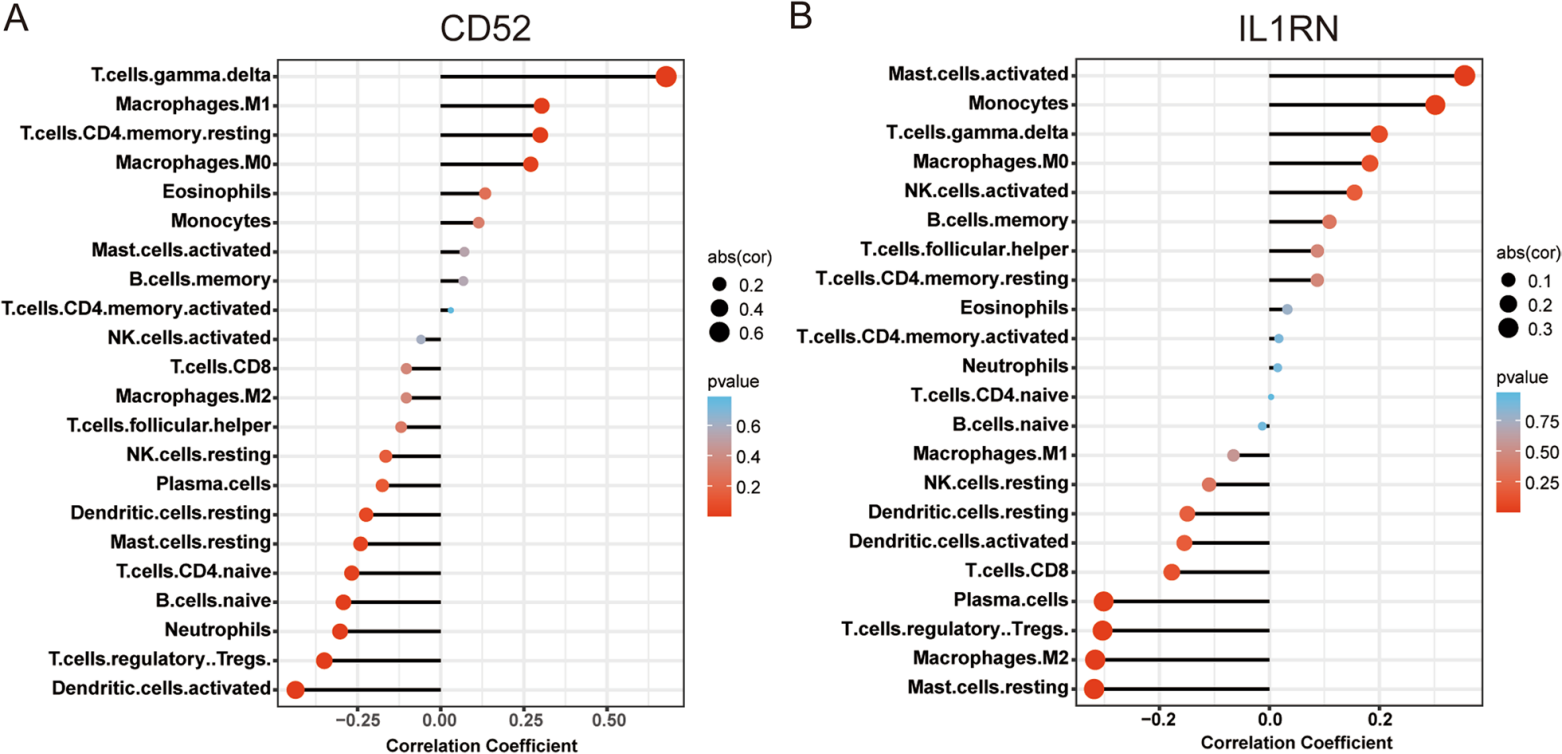
Correlation between hub genes and immune cells. **A** the correlation between CD52 and immune cells. **B** Correlation between IL1RN and immune cells. The size of the points indicates the strength of the correlation, and the color of the points indicates the p-value

The correlation between hub genes and infiltrated immune cells was analyzed by Spearman’s rank correlation in the R software (Additional file 8: Table S8). The results showed that CD52 had a positive correlation with gamma delta T cells, M1 macrophages, CD4 memory resting T cells and M0 macrophages, and a negative correlation with resting dendritic cells, resting mast cells, CD4 naive T cells, naive B cells, Neutrophils, regulatory Tregs T cells and activated dendritic cells (Fig. 8A). Meanwhile, IL1RN had a positive correlation with monocytes and activated mast cells, and a negative correlation with resting mast cells, M2 macrophages, regulatory Tregs T cells and plasma cells (Fig. 8B).

### RT-qPCR validation of the hub genes

**Fig. 9:**
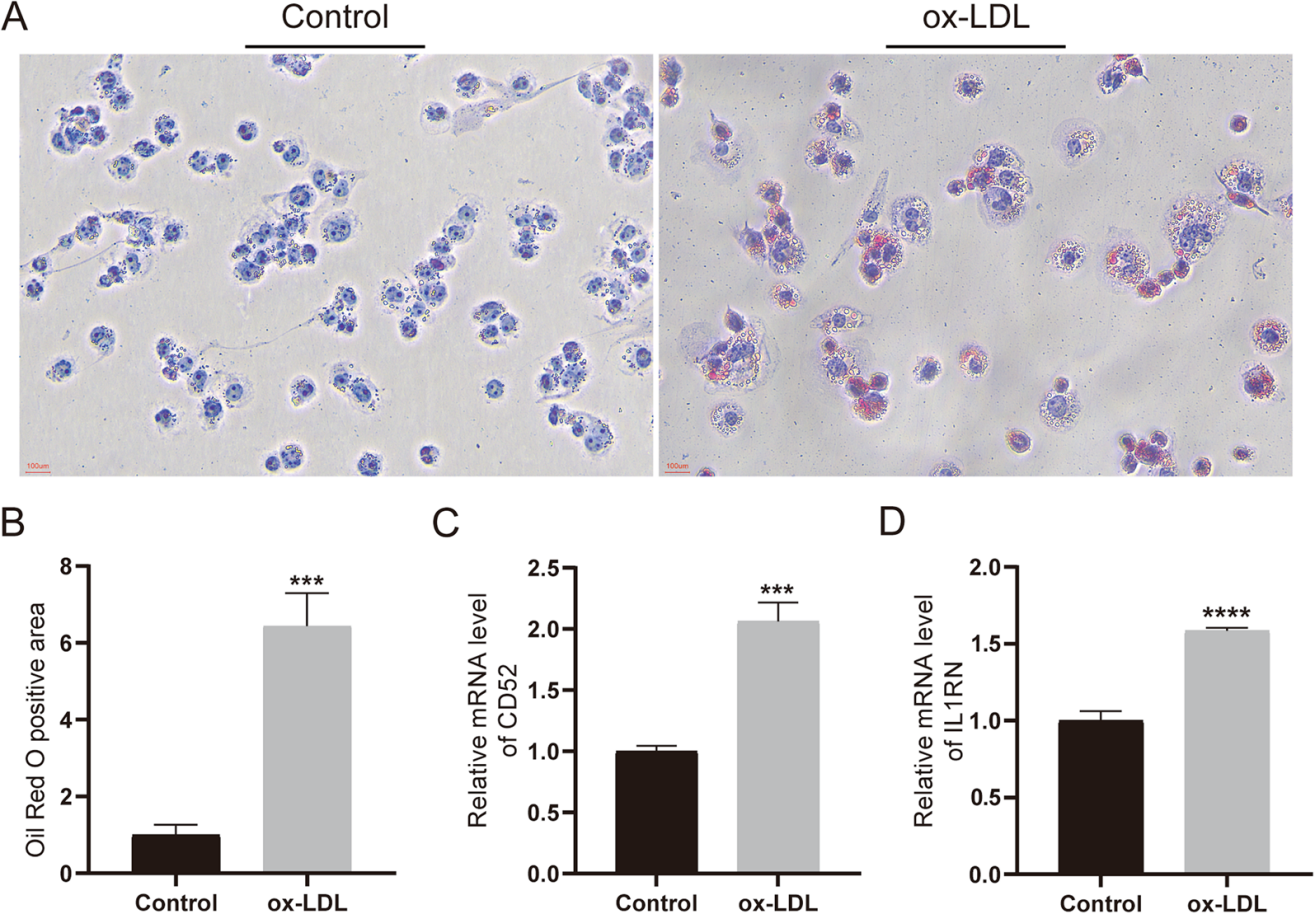
Validation of the CD52 and IL1RN. **A-B** Oil red O staining of cells not treated with ox-LDL and cells treated with ox-LDL; **C-D** The RT-qPCR analysis of the mRNA levels of CD52 and IL1RN in the control group and ox-LDL treatment group

After 48 h of PMA induction, THP-1 became macrophages, which were treated with ox-LDL (80 µg/mL) for an additional 24 h to form foam cells (Fig. 9A). Oil red O staining results showed significantly higher lipid accumulation in ox-LDL-induced foam cells compared to untreated cells (Fig. 9B). We then further detected the expression of CD52 and IL1RN in the atherosclerotic cell model by RT-qPCR. Results showed significantly increased expression of CD52 and IL1RN in ox-LDL-treated cells (Fig. 9C-D), suggesting that CD52 and IL1RN were induced in atherosclerosis cell models.

## Discussion

ASCVD based on atherosclerosis often leads to ischaemic disease with high disability and mortality rates, which can seriously affect the quality of life of patients and impose a heavy burden on society. Despite numerous efforts, the molecular mechanisms of atherosclerosis are still not fully understood. Therefore, there is an urgent need to clarify the pathogenesis of atherosclerosis and find potential diagnostic and therapeutic targets. In recent years, an increasing number of bioinformatics studies have been applied to search for new potential biomarkers of atherosclerosis to explore deeper mechanisms. For example, Wang et al. identified immune cell infiltration and diagnostic biomarkers in unstable atherosclerotic plaques through comprehensive bioinformatics analysis and machine learning [17]. Additionally, Gu et al. used bioinformatics analysis to identify candidate targets for the diagnosis and treatment of atherosclerosis [18], and Tan et al. unveiled the landscape in which immune cell infiltration and immune-related pathways participated in the progression of carotid atherosclerotic plaques through bioinformatics analysis [7].

In this study, we used bioinformatics techniques to identify potential biomarkers in atherosclerosis. The GO enrichment results from our robust DEGs indicated that atherosclerosis was related to the regulation of cytokines, immunity and inflammation, and the enrichment analysis of KEGG pathways also suggested that atherosclerosis was related to the regulation of the chemokine signalling pathway and lipolysis of adipocytes. Indeed, the present study suggests that the occurrence and development of atherosclerosis is strongly related to immunity, inflammation, and lipid metabolism, which is consistent with our results [19, 20]. Additionally, in this study, we identified several potential small molecule compounds that could reverse changes in robust DEGs expression, which might improve atherosclerosis. Vitamin K2 has been reported to have a protective effect against cardiovascular disease, but the role of phylloquinone as vitamin K1 in atherosclerosis is controversial [21, 22]. The effect of desoxypeganine on atherosclerosis has not been studied, but donepezil, which is also an acetylcholinesterase inhibitor, may have a protective effect against atherosclerosis [23]. Similarly, MBCQ has not been reported to treat atherosclerosis, but cilostazol, which is also a phosphodiesterase inhibitor, has a protective effect on atherosclerosis [24, 25]. Other drugs/molecules, including KU-C103428N, gossypol, kavain, flubendazole, VX-702, palonosetron and linsitinib, may be potential antiatherosclerotic agents. Furthermore, by constructing a robust DEGs PPI network and integrating the 12 algorithms of cytoHubba, we identified two key genes, CD52 and IL1RN, and validated their performance in the diagnosis of atherosclerosis. Human CD52 is a glycosylphosphatidylinositol (GPI)-anchored glycoprotein that consists of a 12-amino acid short peptide that is attached to the GPI anchor at its C-terminus and a core fucosylated polylactosamine multiantennal sialosylated glycan at its N- glycosylation site [26–28]. CD52 is widely expressed in immune cells. In maintaining the steady state of the immune system, CD52 is released from its GPI anchors and binds to the inhibitory receptor Siglec-10 [29]. Then, it inhibits the phosphorylation of the T-cell receptor-associated kinases Lck and Zap70, which are required for T-cell antigen receptor signalling, and inhibits the T-cell activation [29]. In monocytes and macrophages, soluble CD52 inhibits NF-κB-mediated signaling and induces apoptosis at higher concentrations [28]. In addition, CD52 plays an important role in autoimmune diseases, such as systemic lupus erythematosus and systemic sclerosis [30, 31]. Alemtuzumab, an anti-CD52 depletion antibody, is already FDA-approved for the treatment of multiple sclerosis and has potential for use in the treatment of acute lymphoblastic leukaemia and myeloid leukaemia [32–34]. ​The role of CD52 in atherosclerosis has not been studied, but atherosclerosis is closely related to immunity and inflammation. Thus, based on the results of the bioinformatics analysis, we speculate that changes in CD52 expression play an important role in atherosclerosis.​.

Interleukin-1 receptor antagonist (IL-1Ra), encoded by the IL1RN gene, is a member of the interleukin-1 (IL-1) family and an endogenous inhibitor of IL-1. It can competitively bind IL-1α and Il-1β to the IL-1 receptor, but does not induce any intracellular response [35]. IL-1Ra can be produced in endothelial cells, smooth muscle cells, and macrophages [36]. It has been shown that the expression level of IL-1Ra is increased in atherosclerotic lesions [37, 38]. Additionally, the absence of IL-1Ra promotes neointimal formation after femoral artery injury in mice and exacerbates atherosclerotic lesion size in ApoE^−/−^ mice [39, 40]. Anakinra is a recombinant human IL-1Ra that acts as a competitive inhibitor of IL-1 by binding to the IL-1 I receptor [41]. Anakinra was shown to reduce plaque size and serum triglycerides in the aortic arch of ApoE^−/−^ mice and to have antiatherosclerotic effects [42]. Current evidence suggests that IL-1Ra plays an important role in atherosclerosis, which is consistent with the results of our bioinformatics analysis. IL-1Ra is also associated with postinfarct cardiac remodelling and heart failure. IL-1Ra has been reported to be an independent predictor of documented adverse outcomes in patients with CAD, particularly in the setting of ACS, and its prognostic value exceed the prognostic values of hs-CRP and troponin T [43]. Studies have shown that anakinra inhibits apoptosis in experimental acute myocardial infarction [44]. The results from pooled analysis of VCUART clinical trials suggest that IL-1 blockade with anakinra for 14 days in STEMI patients can reduce the incidence of new onset HF or HF hospitalizations within 1 year after STEMI surgery [45]. Additionally, the REDHART results indicating improvement in peak oxygen consumption in newly decompensated systolic HF patients after 12 weeks of treatment with anakinra [46]. In addition, Su et al. screened out IL1RN as a possible common pathogenesis of psoriasis and atherosclerosis through bioinformatics analysis, but this finding still needs to be supported by external experimental data [47].

In fact, the pathogenesis of atherosclerosis is very complex, involving many hypotheses and theories, of which “lipid infiltration” and “inflammatory response” are the core. On the one hand, very low-density lipoprotein, low-density lipoprotein and lipoprotein invade and accumulate in the blood vessel wall, and some form oxidized low-density lipoprotein (ox-LDL) [48]. Ox-LDL induces and aggravates the occurrence and development of atherosclerosis. On the other hand, monocytes differentiate into macrophages and engulf a large number of lipids to be converted into foam cells, which secrete a variety of inflammatory factors and promote the development of atherosclerosis [49]. Therefore, foam cells play an important role in all stages of the development of atherosclerotic lesions [50]. In addition, our use of CIBERSORT and Spearman rank correlation analysis suggested that CD52 and IL1RN were strongly associated with monocytes or macrophages. PMA can induce THP-1 cells to transform into macrophages, and ox-LDL can induce macrophages to transform into foam cells, which can be used as a cell model of atherosclerosis to a certain extent [51, 52]. After THP-1 cells were induced to become foam cells, RT‒qPCR detected a significant increase in the mRNA expression levels of CD52 and IL1RN compared to untreated cells. These findings are in full agreement with the results of our bioinformatics analysis, indicating that CD52 and IL1RN are closely related to the occurrence and development of atherosclerosis.

It must be acknowledged that our study has some limitations. First, our study was retrospective, with data from a public database, and lacked data on clinical characteristics. Second, it should be acknowledged that the number of cases in the GSE97210 dataset is insufficient. Finally, the screening of two key genes involved in the occurrence and progression of atherosclerosis is based on bioinformatics techniques. While this indicates that CD52 and IL1RN are highly expressed in foam cells, they still need to be further validated in vitro and in vivo to further explore their potential mechanisms in atherosclerosis.

## Conclusion

​In summary, through the analysis of comprehensive bioinformatics and multiple algorithms, we have identified that CD52 and IL1RN may play a key role in the occurrence and development of atherosclerosis These findings open new lines of thought for further research into the pathogenesis of atherosclerosis.

## Supplementary Information

**Additional file 1. Supplementary Figure S1:** ROC curve evaluation of the diagnostic effectiveness of candidate biomarkers using the GSE43292, GSE97210, and GSE100927 datasets.

**Additional file 2. Supplementary Table S1:** Basic information of selected microarray datasets.

**Additional file 3. Supplementary Table S2:** Differentially expressed genes and their calculated values in the GSE43292, GSE97210, and GSE100927 datasets.

**Additional file 4. Supplementary Table S3:** 155 robust differentially expressed genes identified by RRA method and their calculated values.

**Additional file 5. Supplementary Table S4:** The GO functional enrichment analysis results of 155 robust DEGs.

**Additional file 6. Supplementary Table S5:** The KEGG pathway analysis results of 155 robust DEGs.

**Additional file 7. Supplementary Table S6:** Correlation coefficient of correlation heatmap related to cell type abundance.

**Additional file 8. Supplementary Table S7:** The P-value of correlation coefficient of correlation heatmap related to cell type abundance.

**Additional file 9. Supplementary Table S8:** The correlation between hub genes and infiltrated immune cells.

## Data Availability

The datasets analyzed during the current study are available in the GEO repository (https://www.ncbi.nlm.nih.gov/geo/; Accession numbers: GSE43292, GSE97210, GSE100927 and GSE40231).

## References

[1] Sandesara PB, Virani SS, Fazio S, Shapiro MD (2019). The forgotten lipids: triglycerides, remnant cholesterol, and atherosclerotic Cardiovascular Disease Risk. Endocr Rev.

[2] Herrington W, Lacey B, Sherliker P, Armitage J, Lewington S (2016). Epidemiology of atherosclerosis and the potential to reduce the global burden of Atherothrombotic Disease. Circul Res.

[3] Diseases GBD, Injuries C (2020). Global burden of 369 diseases and injuries in 204 countries and territories, 1990–2019: a systematic analysis for the global burden of Disease Study 2019. Lancet (London England).

[4] Colantonio LD, Shannon ED, Orroth KK, Zaha R, Jackson EA, Rosenson RS, Exter J, Mues KE, Muntner P (2019). Ischemic event rates in very-high-risk adults. J Am Coll Cardiol.

[5] Li B, Xia Y, Hu B (2020). Infection and atherosclerosis: TLR-dependent pathways. Cell Mol Life Sci.

[6] Zhang S, Li L, Chen W, Xu S, Feng X, Zhang L (2021). Natural products: the role and mechanism in low-density lipoprotein oxidation and atherosclerosis. Phytother Res.

[7] Tan L, Xu Q, Shi R, Zhang G (2021). Bioinformatics analysis reveals the landscape of immune cell infiltration and immune-related pathways participating in the progression of carotid atherosclerotic plaques. Artif Cells Nanomed Biotechnol.

[8] Xu J, Zhou H, Cheng Y, Xiang G (2022). Identifying potential signatures for atherosclerosis in the context of predictive, preventive, and personalized medicine using integrative bioinformatics approaches and machine-learning strategies. EPMA J.

[9] Yang R, Yao L, Du C, Wu Y (2021). Identification of key pathways and core genes involved in atherosclerotic plaque progression. Ann Transl Med.

[10] Kolde R, Laur S, Adler P, Vilo J (2012). Robust rank aggregation for gene list integration and meta-analysis. Bioinf (Oxford England).

[11] Kanehisa M, Goto S (2000). KEGG: kyoto encyclopedia of genes and genomes. Nucleic Acids Res.

[12] Yu G, Wang LG, Han Y, He QY (2012). clusterProfiler: an R package for comparing biological themes among gene clusters. OMICS.

[13] Kanehisa M, Furumichi M, Sato Y, Kawashima M, Ishiguro-Watanabe M (2023). KEGG for taxonomy-based analysis of pathways and genomes. Nucleic Acids Res.

[14] Subramanian A, Narayan R, Corsello SM, Peck DD, Natoli TE, Lu X, Gould J, Davis JF, Tubelli AA, Asiedu JK (2017). A Next Generation Connectivity Map: L1000 platform and the first 1,000,000 profiles. Cell.

[15] Szklarczyk D, Gable AL, Nastou KC, Lyon D, Kirsch R, Pyysalo S, Doncheva NT, Legeay M, Fang T, Bork P (2021). The STRING database in 2021: customizable protein-protein networks, and functional characterization of user-uploaded gene/measurement sets. Nucleic Acids Res.

[16] Newman AM, Steen CB, Liu CL, Gentles AJ, Chaudhuri AA, Scherer F, Khodadoust MS, Esfahani MS, Luca BA, Steiner D (2019). Determining cell type abundance and expression from bulk tissues with digital cytometry. Nat Biotechnol.

[17] Wang J, Kang Z, Liu Y, Li Z, Liu Y, Liu J (2022). Identification of immune cell infiltration and diagnostic biomarkers in unstable atherosclerotic plaques by integrated bioinformatics analysis and machine learning. Front Immunol.

[18] Gu Y, Ma X, Li J, Ma Y, Zhang Y (2021). Identification of candidate targets for the diagnosis and treatment of atherosclerosis by bioinformatics analysis. Am J Transl Res.

[19] Esper RJ, Nordaby RA (2019). Cardiovascular events, diabetes and guidelines: the virtue of simplicity. Cardiovasc Diabetol.

[20] Solanki A, Bhatt LK, Johnston TP (2018). Evolving targets for the treatment of atherosclerosis. Pharmacol Ther.

[21] Halder M, Petsophonsakul P, Akbulut AC, Pavlic A, Bohan F, Anderson E, Maresz K, Kramann R, Schurgers L. Vitamin K: Double Bonds beyond Coagulation Insights into Differences between Vitamin K1 and K2 in Health and Disease. Int J Mol Sci 2019, 20(4). 10.3390/ijms20040896 PMC6413124 30791399

[22] Bus K, Szterk A. Relationship between Structure and Biological Activity of Various Vitamin K Forms. Foods 2021, 10(12). 10.3390/foods10123136 PMC8701896 34945687

[23] Zhou S, Li Z, Liu P, Wang S, Zhao J, Zhang G (2020). Donepezil prevents ox-LDL-Induced attachment of THP-1 monocytes to human aortic endothelial cells (HAECs). Chem Res Toxicol.

[24] Katakami N, Kim YS, Kawamori R, Yamasaki Y (2010). The phosphodiesterase inhibitor cilostazol induces regression of carotid atherosclerosis in subjects with type 2 diabetes mellitus: principal results of the Diabetic Atherosclerosis Prevention by Cilostazol (DAPC) study: a randomized trial. Circulation.

[25] Sohn M, Chun EJ, Lim S (2022). Cilostazol treatment for preventing adverse cardiovascular events in patients with type 2 diabetes and coronary atherosclerosis: long-term follow-up of the ESCAPE study. J Diabetes.

[26] Treumann A, Lifely MR, Schneider P, Ferguson MA (1995). Primary structure of CD52. J Biol Chem.

[27] Toh BH, Kyaw T, Tipping P, Bobik A (2013). Immune regulation by CD52-expressing CD4 T cells. Cell Mol Immunol.

[28] Rashidi M, Bandala-Sanchez E, Lawlor KE, Zhang Y, Neale AM, Vijayaraj SL, O’Donoghue R, Wentworth JM, Adams TE, Vince JE (2018). CD52 inhibits toll-like receptor activation of NF-kappaB and triggers apoptosis to suppress inflammation. Cell Death Differ.

[29] Bandala-Sanchez E, Zhang Y, Reinwald S, Dromey JA, Lee BH, Qian J, Bohmer RM, Harrison LC (2013). T cell regulation mediated by interaction of soluble CD52 with the inhibitory receptor Siglec-10. Nat Immunol.

[30] Bhamidipati K, Silberstein JL, Chaichian Y, Baker MC, Lanz TV, Zia A, Rasheed YS, Cochran JR, Robinson WH (2020). CD52 is elevated on B cells of SLE patients and regulates B cell function. Front Immunol.

[31] Rudnik M, Rolski F, Jordan S, Mertelj T, Stellato M, Distler O, Blyszczuk P, Kania G (2021). Regulation of Monocyte Adhesion and Type I Interferon Signaling by CD52 in patients with systemic sclerosis. Arthritis Rheumatol.

[32] Willis MD, Robertson NP (2016). Alemtuzumab for multiple sclerosis. Curr Neurol Neurosci Rep.

[33] Wei G, Wang J, Huang H, Zhao Y (2017). Novel immunotherapies for adult patients with B-lineage acute lymphoblastic leukemia. J Hematol Oncol.

[34] Karnan S, Hanamura I, Ota A, Takasugi S, Nakamura A, Takahashi M, Uchino K, Murakami S, Wahiduzzaman M, Quang Vu L (2021). CD52 is a novel target for the treatment of FLT3-ITD-mutated myeloid leukemia. Cell Death Discov.

[35] Arend WP, Malyak M, Guthridge CJ, Gabay C (1998). Interleukin-1 receptor antagonist: role in biology. Annu Rev Immunol.

[36] Fearon WF, Fearon DT (2008). Inflammation and cardiovascular disease: role of the interleukin-1 receptor antagonist. Circulation.

[37] Dewberry R, Holden H, Crossman D, Francis S (2000). Interleukin-1 receptor antagonist expression in human endothelial cells and atherosclerosis. Arterioscler Thromb Vasc Biol.

[38] Olofsson PS, Sheikine Y, Jatta K, Ghaderi M, Samnegard A, Eriksson P, Sirsjo A (2009). A functional interleukin-1 receptor antagonist polymorphism influences atherosclerosis development. The interleukin-1beta:interleukin-1 receptor antagonist balance in atherosclerosis. Circ J.

[39] Isoda K, Shiigai M, Ishigami N, Matsuki T, Horai R, Nishikawa K, Kusuhara M, Nishida Y, Iwakura Y, Ohsuzu F (2003). Deficiency of interleukin-1 receptor antagonist promotes neointimal formation after injury. Circulation.

[40] Isoda K, Sawada S, Ishigami N, Matsuki T, Miyazaki K, Kusuhara M, Iwakura Y, Ohsuzu F (2004). Lack of interleukin-1 receptor antagonist modulates plaque composition in apolipoprotein E-deficient mice. Arterioscler Thromb Vasc Biol.

[41] Abbate A, Toldo S, Marchetti C, Kron J, Van Tassell BW, Dinarello CA (2020). Interleukin-1 and the Inflammasome as therapeutic targets in Cardiovascular Disease. Circul Res.

[42] Ku EJ, Kim BR, Lee JI, Lee YK, Oh TJ, Jang HC, Choi SH. The Anti-Atherosclerosis Effect of Anakinra, a Recombinant Human Interleukin-1 Receptor Antagonist, in Apolipoprotein E Knockout Mice. Int J Mol Sci 2022, 23(9). 10.3390/ijms23094906 PMC9104865 35563294

[43] Schofer N, Ludwig S, Rubsamen N, Schnabel R, Lackner KJ, Ruprecht HJ, Bickel C, Landmesser U, Blankenberg S, Zeller T (2018). Prognostic impact of Interleukin-1 receptor antagonist in patients with documented coronary artery disease. Int J Cardiol.

[44] Abbate A, Salloum FN, Vecile E, Das A, Hoke NN, Straino S, Biondi-Zoccai GG, Houser JE, Qureshi IZ, Ownby ED (2008). Anakinra, a recombinant human interleukin-1 receptor antagonist, inhibits apoptosis in experimental acute myocardial infarction. Circulation.

[45] Abbate A, Wohlford GF, Del Buono MG, Chiabrando JG, Markley R, Turlington J, Kadariya D, Trankle CR, Biondi-Zoccai G, Lipinski MJ (2022). Interleukin-1 blockade with anakinra and heart failure following ST-segment elevation myocardial infarction: results from a pooled analysis of the VCUART clinical trials. Eur Heart J Cardiovasc Pharmacother.

[46] Van Tassell BW, Canada J, Carbone S, Trankle C, Buckley L, Oddi Erdle C, Abouzaki NA, Dixon D, Kadariya D, Christopher S et al. Interleukin-1 Blockade in Recently Decompensated Systolic Heart Failure: Results From REDHART (Recently Decompensated Heart Failure Anakinra Response Trial). Circ Heart Fail 2017, 10(11). 10.1161/CIRCHEARTFAILURE.117.004373 PMC5699505 29141858

[47] Su W, Zhao Y, Wei Y, Zhang X, Ji J, Yang S (2021). Exploring the pathogenesis of Psoriasis Complicated with atherosclerosis via microarray data analysis. Front Immunol.

[48] Khatana C, Saini NK, Chakrabarti S, Saini V, Sharma A, Saini RV, Saini AK. Mechanistic Insights into the Oxidized Low-Density Lipoprotein-Induced Atherosclerosis. *Oxidative medicine and cellular longevity* 2020, 2020:5245308. 10.1155/2020/5245308 PMC7512065 33014272

[49] Mo C, Yang M, Han X, Li J, Gao G, Tai H, Huang N, Xiao H (2017). Fat mass and obesity-associated protein attenuates lipid accumulation in macrophage foam cells and alleviates atherosclerosis in apolipoprotein E-deficient mice. J Hypertens.

[50] Barrett TJ (2020). Macrophages in atherosclerosis regression. Arterioscler Thromb Vasc Biol.

[51] Bekkering S, Quintin J, Joosten LA, van der Meer JW, Netea MG, Riksen NP (2014). Oxidized low-density lipoprotein induces long-term proinflammatory cytokine production and foam cell formation via epigenetic reprogramming of monocytes. Arterioscler Thromb Vasc Biol.

[52] Li L, Du Z, Rong B, Zhao D, Wang A, Xu Y, Zhang H, Bai X, Zhong J. Foam cells promote atherosclerosis progression by releasing CXCL12. Biosci Rep 2020, 40(1). 10.1042/BSR20193267 PMC6970083 31894855

