# Supplementary material for: Identification of potential diagnostic biomarkers of atherosclerosis based on bioinformatics strategy
Source: BMC Med Genomics. 2023 May 12;16:100. doi: 10.1186/s12920-023-01531-w (PMC10176947; doi:10.1186/s12920-023-01531-w)
Supplement: Supplementary file 1 — Additional file 1. Supplementary Figure S1: ROC curve evaluation of the diagnostic effectiveness of candidate biomarkers using the GSE43292, GSE97210, and GSE100927 datasets. [file 12920_2023_1531_MOESM1_ESM.docx]

**Supplementary Table S1:** Basic information of selected microarray datasets.

| GEO | Author/Reference | Platform | Samples (number) | | Attribute |
| --- | --- | --- | --- | --- | --- |
|  |  |  | Atherosclerotic artery | Control artery |  |
| GSE43292 | G. Bricca | GPL6244 | 32 | 32 | Test |
| GSE97210 | Y. Hu | GPL16956 | 3 | 3 | Test |
| GSE100927 | M. Steenman | GPL17077 | 29(carotid) | 12(carotid) | Test |
| GSE40231 | S. Hägg | GPL570 | 40 | 40 | Validation |
