# Supplementary material for: Identification of potential diagnostic biomarkers of atherosclerosis based on bioinformatics strategy
Source: BMC Med Genomics. 2023 May 12;16:100. doi: 10.1186/s12920-023-01531-w (PMC10176947; doi:10.1186/s12920-023-01531-w)
Supplement: Supplementary file 9 — Additional file 9. Supplementary Table S8: The correlation between hub genes and infiltrated immune cells. [file 12920_2023_1531_MOESM9_ESM.docx]

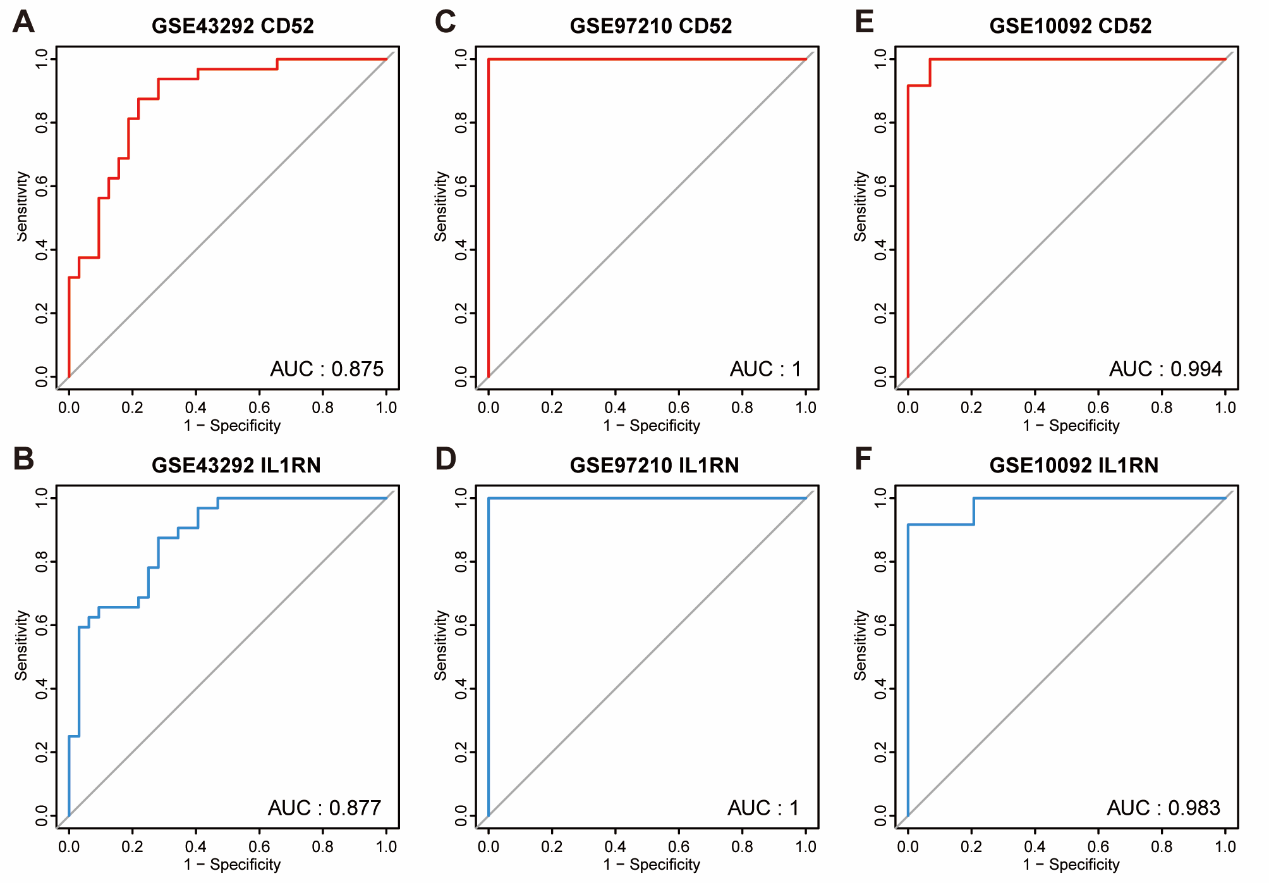


**Supplementary Figure S1** ROC curve evaluation of the diagnostic effectiveness of candidate biomarkers using the GSE43292, GSE97210, and GSE100927 datasets. (**A**, **C, E**) The diagnostic value of CD52 for atherosclerosis in the GSE43292, GSE97210, and GSE100927 datasets. (**B, D, F**) The diagnostic value of IL1RN for atherosclerosis in the GSE43292, GSE97210, and GSE100927 datasets.
